# Comparison of Protective Effects of Shenmai Injections Produced by Medicinal Materials from Different Origins on Cardiomyocytes

**DOI:** 10.1155/2022/7205476

**Published:** 2022-03-18

**Authors:** Min Zhao, Qiufang Chen, Hong Wang, Wanfeng Xu, Jiayong Yang, Lijuan Cao

**Affiliations:** ^1^Department of Pharmacy, The First Affiliated Hospital of Xiamen University, School of Medicine, Xiamen University, Xiamen 361003, China; ^2^Ministry of Science and Education, Women and Children's Hospital, School of Medicine, Xiamen University, Xiamen 361003, China; ^3^State Key Laboratory of Natural Medicines, Key Lab of Drug Metabolism and Pharmacokinetics, China Pharmaceutical University, Nanjing 211198, China

## Abstract

Shenmai injection is mainly used for the treatment of heart-related diseases, including coronary heart disease, viral myocarditis, chronic cor pulmonale, and shock in Asia. Medicinal materials from different origins produce Shenmai injections for clinical use, and their protective effects on cardiomyocytes may vary with the choice of raw materials. In this study, we compared the protective effects of Shenmai injections produced from different raw materials on cardiomyocytes. Results showed that the protective effects of various Shenmai injections on hypoxia-reoxygenation-induced cardiomyocyte injury were mainly attributed to total ginsenosides extract, with few differences between them. However, the protective effects of different Shenmai injections on doxorubicin and oxidative stress-induced cardiomyocyte injury were significantly different; the protective effects of Shenmai injection with Zhejiang *Ophiopogon japonicus* as raw material were significantly better than those with Sichuan *Ophiopogon japonicus*, consistent with our previous research results. Our study reveals the different cardiomyocyte protective effects of Shenmai injections produced by medicinal materials from different origins, laying a scientific foundation for their clinical selection.

## 1. Introduction

Cardiovascular diseases are a global health problem that is becoming an increasingly significant cause of death. Many cardiovascular diseases are accompanied by cardiomyocyte ischemia and oxidative stress, leading to cardiomyocyte injury [1]. As adult cardiomyocytes irreversibly withdraw from the cell cycle soon after birth, it is hard for them to proliferate and regenerate after injury [2, 3]. Cardiomyocyte injury is a common serious and intractable complication in patients with cardiovascular diseases. Therefore, cardioprotection is particularly important during treatment. Cardioprotective strategies fall into four broad categories, which can be combined to achieve multitarget cardioprotection. The main mechanisms are the activation of endogenous prosurvival pathways and inhibition of cell death pathways, in which reactive oxygen species (ROS) play an important role [4–6].

Shenmai injection is a widely used traditional Chinese medicine injection, which is extracted and refined from red ginseng (*Panax ginseng* C. A. Meyer) and *Ophiopogon japonicus* (Linn. f.) Ker-Gawl (*O. japonicas,* Maidong in Chinese). Its main active components include various ginsenosides, *O. japonicas* saponins, and *O. japonicas* flavones. It has the functions of tonifying Qi and preventing exhaustion, nourishing Yin, and promoting body fluid, which could regulate immunity, strengthen the cardiovascular system, and provide resistance against inflammation and oxidization with few adverse reactions [7]; it is mainly used to treat shock, coronary heart disease, viral myocarditis, chronic cor pulmonale, neutropenia, etc. [8]. Clinically, it has protective effects on cardiomyocyte injury induced by various factors [9, 10]. Shenmai injection can also improve the heart function classification of patients with chronic heart failure and coronary artery disease, according to the New York Heart Association [11].

*O. japonicas* is a traditional Chinese medicinal herb commonly used in Asia. The raw materials of *O. japonicas* in Shenmai injection are authentic. *O. japonicas* extract has many pharmacological activities, including cardiovascular protection, antioxidant properties, anticancer properties, immune regulation, and diabetes treatment [12–15]. The components of *O. japonicas* extract are complex, according to comprehensive reports. More than 75 kinds of steroidal saponins, 36 kinds of *O. japonicas* flavonoids, 11 kinds of polysaccharides, and 13 kinds of organic acids have been isolated and identified [16]. *O. japonicas* is mainly planted in the Sichuan and Zhejiang provinces in China, respectively called Chuan maidong and Zhe maidong. Their water content, ash content, flavone and polysaccharide components, and other characteristics differ significantly, indicating that different planting methods and production areas influence the characteristics of *O. japonicas*. Previous research in our laboratory shows significant differences in the active components and efficacy of Sichuan and Zhejiang *O. japonicas* extracts. These results have a high reference value for the resource development and clinical application of *O. japonicas* [17].

Different manufacturers use *O. japonicas* from different origins to produce Shenmai injections for clinical use. Most manufacturers use Sichuan *O. japonicas* as a raw material; only ChiaTai Qingchunbao Pharmaceutical Co., Ltd. Uses Zhejiang *O. japonicas* as raw material. As raw materials have a crucial impact on the quality of Chinese patent medicine, whether there are differences in cardiomyocyte protective effects of these Shenmai injections is unknown and deserves further research. In this study, we examined the different cardiomyocyte protective effects of various Shenmai injections, providing references for their clinical selection.

## 2. Materials and Methods

### 2.1. Materials, Reagents, and Chemicals

Shenmai injections are produced by five manufacturers; Sichuan and Zhejiang *O. japonicas* extracts were obtained from ChiaTai Qingchunbao Pharmaceutical Co., Ltd. (Hangzhou, China). The first four Shenmai injections (indicated as SM1–4) were made from Sichuan *O. japonicas,* and the fifth Shenmai injection (indicated as SM5) was made from Zhejiang *O. japonicas*. The total ginsenosides extract was purchased from the College of Chemistry of Jilin University (Changchun, China). H_2_O_2_ was purchased from Sigma-Aldrich (Shanghai, China). A cell counting kit-8 (CCK-8) was purchased from DOJINDO (Kumamoto, Japan). An annexinV-FITC/PI apoptosis detection kit was purchased from BD Biosciences (CA, USA). A lactate dehydrogenase (LDH) release assay kit, a ROS assay kit, a BCA protein assay kit, N-acetylcysteine (NAC), and resveratrol were purchased from Beyotime (Shanghai, China).

### 2.2. Extracts Preparation

A total of 200 g *O. japonicas* were cut into pieces and soaked with 500 ml 75% ethanol for 15 min at 20°C. Then, the mixture was heat reflux extracted for 2 h twice in 500 ml 75% ethanol. The ethanol extract was collected and condensed to 500 ml under reduced pressure. Extracts were lyophilized and redissolved with an equal volume of Dulbecco's modified Eagle medium (DMEM) for cell experiments.

### 2.3. Cell Culture

Rat cardiomyocytes H9c2 cell line was obtained from American Type Culture Collection (ATCC, Virginia, USA) and grown in DMEM with 10% fetal bovine serum, 100 U/ml penicillin, and 100 *μ*g/ml streptomycin at 37°C in a humidified incubator with 5% CO_2_.

### 2.4. Establishment of Hypoxia-Reoxygenation Model of Cardiomyocytes

H9c2 cells were cultured with mediums containing different concentrations of glucose in a humidified incubator with 5% CO_2_ and 95% N_2_ for 24 h, after which cells were cultured in 5% CO_2_ and 95% air for another 3 h. Then, cell viability was detected using CCK-8 assays. In this hypoxia-reoxygenation model, the cell survival rate was about 50%, essentially meeting the requirements of the following experiments.

### 2.5. Cell Viability Assay

Cells in a 96-well plate were treated with agents for an indicated time, and the medium was collected for LDH release assay. 100 *μ*l fresh medium containing 10% CCK-8 agent was added to each well for a further 0.5 h at 37°C. Absorbance at 450 nm was measured using a BioTek Synergy H1 Hybrid Reader (VT, USA).

### 2.6. LDH Release Assay

A total of 120 *μ*l of the CCK-8 assay medium was collected in a new 96-well plate. Then, 60 *μ*l of the working solution provided in the LDH release assay kit was added to each well, and the plate was incubated for a further 0.5 h at 37°C. Absorbance at 490 nm was measured.

### 2.7. ROS Assay

The intracellular ROS levels were detected using a ROS assay kit. Briefly, cells in a 12-well plate were treated with agents for an indicated time, after which the culture medium was discarded. Then, 1 ml of serum-free medium containing a 10 *μ*M ROS probe was added, and cells were incubated for a further 0.5 h at 37°C. After washing with PBS three times, cells were lysed with 300 *μ*l NaOH:CH_3_OH (*V*:*V* = 1 : 1). The fluorescence at 488 nm excitation and 525 nm emission of the cell lysate supernatant was measured.

### 2.8. Cell Apoptosis Assay

H9c2 cells were exposed to doxorubicin or H_2_O_2_ with agents for an indicated time. Apoptosis was analyzed using an annexinV-FITC/PI apoptosis detection kit. The proportion of apoptotic cells in 1 × 10^4^ labeled cells was quantified with Agilent NoveCyte flow cytometer (CA, USA).

### 2.9. Statistical Analysis

Data are representative of three independent experiments and were presented as mean ± S.E.M. (*n* = 3 or 6). Statistical differences were evaluated by a two-tailed Student's *t*-test or one-way analysis of variance (ANOVA). *P* < 0.05 was accepted as statistically significant.

## 3. Results

### 3.1. Protective Effect of Shenmai Injection on Hypoxia-Reoxygenation-Induced Cardiomyocyte Injury Was Mainly Attributed to Total Ginsenosides Extract

**Figure 1:**
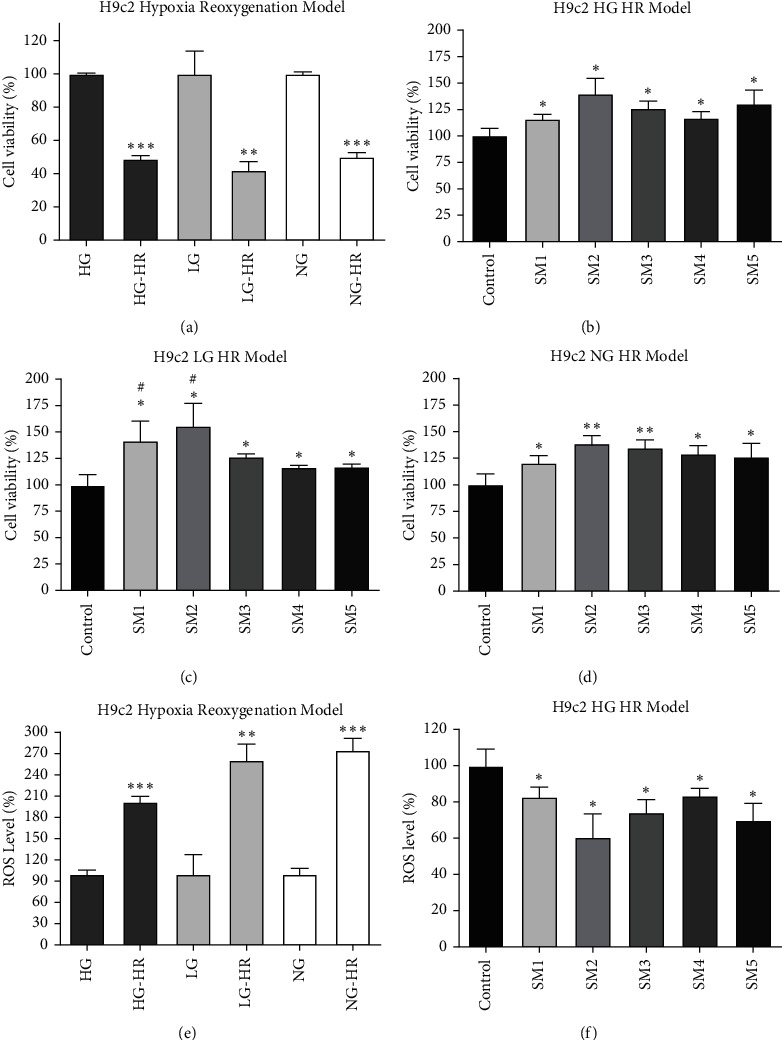
Protective effects of Shenmai injections on cardiomyocyte injury induced by hypoxia-reoxygenation. (a) H9c2 cells were cultured with mediums containing different concentrations of glucose (HG, high glucose, mediums with 4.5 g/L glucose; LG, low glucose, mediums with 1 g/L glucose; NG, no glucose, mediums with 0 g/L glucose) in a humidified incubator with 5% CO_2_ and 95% N_2_ for 24 h after which cells were cultured in 5% CO_2_ and 95% air for another 3 h. Then, cell viability was detected using CCK-8 assays (HR, hypoxia-reoxygenation). (b–d) Cytoprotective effects of 1% ((V):V) Shenmai injections in different models. (e, f) ROS levels of a, b. ^*∗*^*P* < 0.05, ^*∗∗*^*P* < 0.01, ^*∗∗∗*^*P* < 0.001 vs corresponding control groups, Student's *t*-test. ^#^*P* < 0.05 vs SM5 group, Student's *t*-test.

Cardiomyocyte ischemia is the physiological basis of angina pectoris, myocardial infarction, and other heart diseases. Blood reperfusion plays an important role in the recovery of cardiomyocytes. However, the reperfusion of ischemic tissue produces significant ROS in mitochondria, leading to oxidative damage, cell death, and abnormal immune response [18]. The *in vitro* cardiomyocyte hypoxia-reoxygenation model is a suitable simulation of the ischemia-reperfusion of cardiomyocytes *in vivo* [19, 20]. Therefore, we investigated the anti-hypoxia-reoxygenation-induced cell injury effects of Shenmai injections produced by medicinal materials from different origins on rat cardiomyocytes cultured *in vitro*. We first explored the model conditions of hypoxia-reoxygenation injury on a rat cardiomyocyte H9c2 cell line. The results showed that hypoxia for 24 h and reoxygenation for another 3 h met the requirements of cell injury under high glucose (4.5 g/L), low glucose (1g/L), and glucose-free (0g/L) conditions. The cell survival results are shown in Figure 1(a). We next tested the protective effects of five kinds of Shenmai injections on hypoxia-reoxygenation-induced cardiomyocyte injury in high glucose, low glucose, and glucose-free mediums. Results showed that Shenmai injections could alleviate the cardiomyocyte injury caused by hypoxia-reoxygenation in different degrees (Figures 1(b)–1(d)). We also measured the ROS production of cells under hypoxia-reoxygenation; results are shown in Figures 1(e) and 1(f). Although a large number of ROS were produced during hypoxia-reoxygenation, Shenmai injections significantly reduced intracellular ROS levels and effectively reversed hypoxia-reoxygenation-induced cardiomyocyte injury.

**Figure 2:**
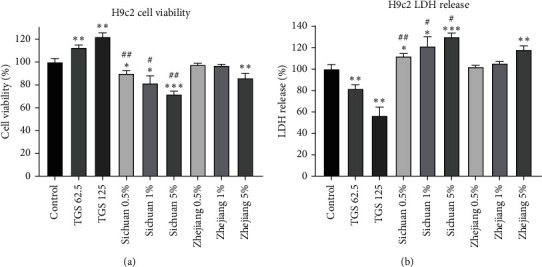
Protective effects of single drug extract from Shenmai injections on hypoxia-reoxygenation-induced cardiomyocyte injury. (a) Cardiomyocytes were treated with total ginsenosides extract or 0.5%–5% (V:V) O. japonicas extracts during hypoxia-reoxygenation (low glucose); cell viability was detected using the CCK-8 assay (TGS 62.5, TGS 125 : 62.5 and 125 *μ*g/ml total ginsenosides extract). (b) LDH release experiment of a. ^*∗*^*P* < 0.05, ^*∗∗*^*P* < 0.01, ^*∗∗∗*^*P* < 0.001 vs corresponding control group, Student's *t*-test. ^#^*P* < 0.05, ^##^*P* < 0.01 vs the same concentration of Zhejiang *O. japonicas* extract groups, Student's *t*-test.

In the hypoxia-reoxygenation-induced cardiomyocyte injury model, five Shenmai injections had significant protective effects. According to the one-way ANOVA (see Supplementary Files), there were no significant differences between five Shenmai injections in Figures 1(b) and 1(d); however, although there were significant differences in Figures 1(c) and 1(f), the overall differences were small and not significant. We then investigated the protective effects of total ginsenosides extract and Sichuan and Zhejiang *O. japonicas* extracts on H9c2 cells. The results revealed that only total ginsenosides extract could significantly increase cell survival fraction, while Sichuan and Zhejiang *O. japonicas* extracts had little efficacy (Figure 2(a)). The results of the LDH release assay were consistent with cell viability assay; namely, neither Sichuan nor Zhejiang *O. japonicas* extracts could alleviate the increase of lactate dehydrogenase release induced by hypoxia-reoxygenation, and only total ginsenosides extract could reduce its increase (Figure 2(b)). These results indicated that the protective effect of Shenmai injections on hypoxia-reoxygenation-induced cardiomyocyte injury was mainly attributed to total ginsenosides extract.

### 3.2. Shenmai Injection with Zhejiang *O. japonicas* as Raw Material had a Better Protective Effect on Doxorubicin-Induced Cardiomyocyte Injury than Sichuan *O. japonicas*

**Figure 3:**
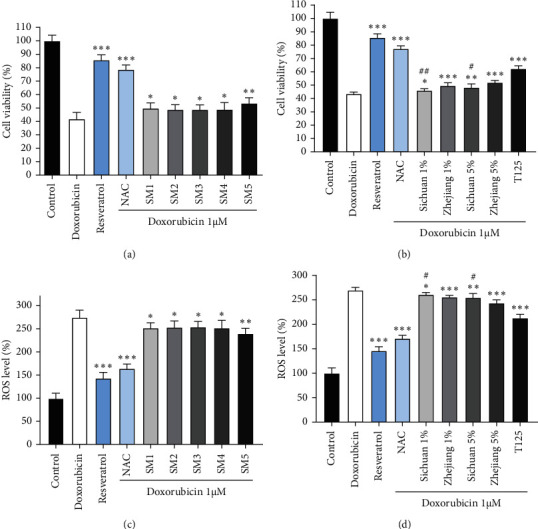
Protective effects of Shenmai injections and their main components on doxorubicin-induced cardiomyocyte injury. (a) Survival rates of H9c2 cells treated with 1 *μ*M doxorubicin combined with 1% (V:V) Shenmai injections for 24 h (resveratrol 100 *μ*M, NAC: acetylcysteine 5 mM). (b) The survival rate of H9c2 cells treated with 1 *μ*M doxorubicin combined with 1% and 5% *O. japonicus* extracts for 24 h (Chuan 1%: medium containing 1% (V:V) Sichuan *O. japonicus* extract, etc.; T125 : 125 *μ*g/ml total ginsenosides extract). (c, d) ROS levels of a, b. ^*∗*^*P* < 0.05, ^*∗∗*^*P* < 0.01, ^*∗∗∗*^*P* < 0.001 vs doxorubicin alone group. ^#^*P* < 0.05, ^##^*P* < 0.01 vs the same concentration of Zhejiang *O. japonicas* extract groups, Student's *t*-test.

**Figure 4:**
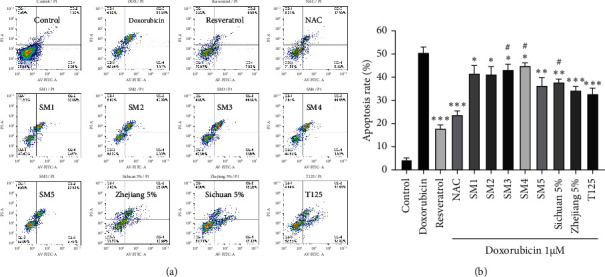
Protective effects of Shenmai injections and their main components on doxorubicin-induced cardiomyocyte apoptosis. (a) Apoptosis rates of H9c2 cells treated with 1 *μ*M doxorubicin combined with 1% Shenmai injections, 5% *O. japonicus* extracts, and 125 *μ*g/ml total ginsenosides extract for 24 h (resveratrol 100 *μ*M, NAC: acetylcysteine 5 mM). (b) Quantitative analysis of a. ^*∗*^*P* < 0.05, ^*∗∗*^*P* < 0.01, ^*∗∗∗*^*P* < 0.001 vs doxorubicin alone group. ^#^*P* < 0.05 vs SM5 or Zhejiang *O. japonicas* extract 5% group, Student's *t*-test.

Clinically, the combination of chemotherapeutic drugs with Shenmai injection can enhance the efficacy of chemotherapeutic drugs and reduce their adverse reactions [21]. The most serious clinical adverse reaction of anthracyclines is their cardiotoxicity, which can induce cardiomyocyte injury and apoptosis. One of its main mechanisms is that anthracyclines can induce cardiomyocytes to produce a large amount of ROS [22]. We next investigated the protective effects of Shenmai injections on the doxorubicin-induced cardiomyocyte injury model *in vitro*. The results are shown in Figure 3(a). Five Shenmai injections could protect cardiomyocyte injury induced by doxorubicin to different degrees. The fifth Shenmai injection with Zhejiang *O. japonicas* as raw material showed superior efficacy than other Shenmai injections. Therefore, we compared the protective effects of Sichuan and Zhejiang *O. japonicas* extracts on this model. According to the results in Figure 3(b), both Sichuan and Zhejiang *O. japonicas* extracts could significantly reduce the death of H9c2 cells exposed to doxorubicin. At the same time, the protective effect of Zhejiang *O. japonicas* extract was better than Sichuan *O. japonicas* extract. We investigated the effect of Shenmai injections on doxorubicin-induced cardiomyocyte apoptosis. It could be seen in Figure 4 that the fifth Shenmai injection had a better protective effect on cardiomyocyte apoptosis caused by doxorubicin. Although the one-way ANOVA of five Shenmai injections in Figure 3 showed no significant difference in their protective effects, the one-way ANOVA of Figure 4 showed significant differences in protecting cardiomyocyte apoptosis induced by doxorubicin; the efficacy of SM5 was better (See Supplementary Files). In summary, these results showed that Shenmai injections may have the potential of alleviating cardiomyocyte injury caused by anthracyclines in chemotherapy patients, whereas Shenmai injections with Zhejiang *O. japonicas* as raw material might be a better choice.

### 3.3. Shenmai Injection with Zhejiang *O. japonicas* as Raw Material had a Better Protective Effect on Oxidative Damage of Cardiomyocytes than Sichuan *O. japonicas*

**Figure 5:**
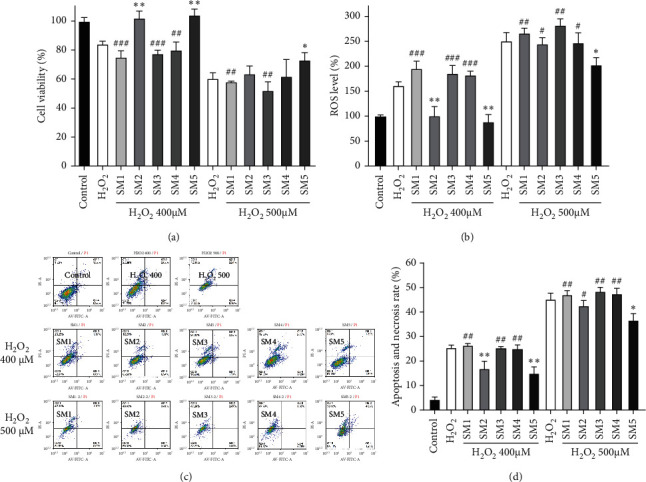
Protective effect of Shenmai injections on cardiomyocyte injury induced by H_2_O_2_. (a) H9c2 cells were treated with 400 or 500 *μ*M H_2_O_2_ with or without 1% (V:V) Shenmai injections for 3 h; cell viability was detected using the CCK-8 assay. (b) ROS level of a. (c) Apoptosis and necrosis rates of H9c2 cells treated with H_2_O_2_ combined with 1% Shenmai injections for 3 h. (d) quantitative analysis of c. ^*∗*^*P* < 0.05, ^*∗∗*^*P* < 0.01 vs the same concentration of H_2_O_2_ groups, ^#^*P* < 0.05, ^##^*P* < 0.01, ^###^*P* < 0.001 vs SM5 groups with the same concentration of H_2_O_2_, Student's *t*-test.

As cardiomyocyte injury induced by ischemia-reperfusion, anthracycline chemotherapy, and other factors are related to ROS, we next established an oxidative stress model with hydrogen peroxide on the H9c2 cell line *in vitro* and investigated the antioxidative stress effect of Shenmai injections produced by medicinal materials from different origins. The results are shown in Figures 5(a) and 5(b). The second and fifth Shenmai injections showed suitable antioxidant effects when H9c2 cells were treated with 400 *μ*M H_2_O_2_ for 3 h, while other Shenmai injections had little efficacy. However, when the dosage of H_2_O_2_ increased to 500 *μ*M, only the fifth Shenmai injection showed significant antioxidant activity. The results of antiapoptosis experiments were consistent with the results of cytotoxicity experiments (Figures 5(c) and 5(d)). The one-way ANOVA of Figure 5 showed that five Shenmai injections had significant differences in protecting oxidative damage of cardiomyocytes; the efficacy of SM5 was better (see the Supplementary Files). It could be inferred that the anti-oxidant effect of Shenmai injection with Zhejiang *O. japonicas* as raw materials was better than that of the other four Shenmai injections with Sichuan *O. japonicas* as raw materials, consistent with our previous research results [17].

## 4. Discussion

Cardiovascular diseases such as coronary heart disease, angina pectoris, and viral myocarditis are often accompanied by cardiomyocyte injury. The main causes include myocardial hypoxia, inflammation, and the accumulation of ROS [23]. In addition, some chemotherapeutic drugs, such as anthracycline, can also cause cardiomyocyte injury [24]. However, there are few suitable clinical treatments to alleviate cardiomyocyte injury [25, 26]. Traditional Chinese medicine has unique advantages in regulating oxidative stress and cell injury. Shenmai injections are a commonly used drug for myocardial protection in clinics, with confirmed efficacy [27]. Many studies have shown that Shenmai injection can alleviate myocardial cell damage caused by many factors, including ischemia-reperfusion, doxorubicin, and hydrogen peroxide. Its specific mechanism involves mitochondrial dynamics modulation, energy metabolism, autophagy regulation, and signal pathway regulation [10, 19, 28–30].

*O. japonicas* have many pharmacological effects related to the cardiovascular field, such as scavenging free radicals, immune regulation, alleviating myocardial ischemia, and arrhythmia. [16]. Ophiopogon polysaccharide, steroidal saponins, and ophiopogonin D can alleviate the injury of cardiomyocytes; its specific mechanism involves inhibiting oxidative stress, regulating inflammatory response, and mitochondrial dynamics [12, 31, 32]. The choice of raw materials of traditional Chinese medicine is very particular, with a significant impact on the efficacy of Chinese patent medicine. Our previous research showed that the total amount of ophiopogonone component in Zhejiang *O. japonicas* extract was 3.16 times that in Sichuan *O. japonicas* extract. Ophiopogonanone B, methyl-ophiopogonanone A, and ophiopogonanone D, among the most important bioactive components of *O. japonicas* extract, were 5–50 times more abundant in Zhejiang *O. japonicas* extract than in Sichuan *O. japonicas* extract. Although the sum of the content of the total ophiopogonins in the two *O. japonicas* extracts did not differ significantly, the ophiopogonin compositions in the two *O. japonicas* extracts showed a significant difference and may be used as unique markers to distinguish the two *O. japonicas* [17]. The active components of Sichuan and Zhejiang *O. japonicas* have noticeable differences, resulting in certain differences in their efficacy and clinical selection of Shenmai injection.

In this study, we investigated the protective effects of Shenmai injections produced by medicinal materials from different origins on cardiomyocyte injury induced by various factors and preliminarily explored the possible mechanisms. We conducted one-way ANOVA on the efficacy comparison of five Shenmai injections in this study. Results showed that Shenmai injections had a certain effect on cardiomyocyte injury induced by hypoxia-reoxygenation. Their efficacy was mainly attributed to total ginsenosides extract, while *O. japonicas* extracts had little effect. The main reason may be that the mechanism of hypoxia-reoxygenation-induced cardiomyocyte injury involves ROS and other mechanisms, such as inflammation, apoptosis, and energy metabolism [33, 34]. Much literature has shown that ginsenosides have significant regulatory effects in these aspects [35]. Therefore, the effects of anti-hypoxia-reoxygenation-induced cardiomyocytes in Shenmai injections produced by medicinal materials from different origins have few differences.

Shenmai injection also had a significant protective effect on cardiomyocyte injury induced by doxorubicin and H_2_O_2_. The efficacy of Shenmai injection with Zhejiang *O. japonicas* as raw material was significantly better than that of Shenmai injections with Sichuan *O. japonicas*. Our previous studies showed that there were significant differences in the components of Zhejiang and Sichuan *O. japonicas* extracts; the anti-inflammatory and oxidative capacity of Zhejiang *O. japonicas* extract were significantly higher than that of Sichuan *O. japonicas* extract. These results are due to the higher amounts of ophiopogonones in Zhejiang *O. japonicas* extract [17]. Our study reveals the differences in cardiomyocyte protection in Shenmai injections produced by medicinal materials from different origins, providing a basis for their clinical selection.

## 5. Conclusion

In summary, the results of this study showed that the protective effect of various Shenmai injections on cardiomyocyte hypoxia-reoxygenation injury is mainly attributed to total ginsenosides extract, with few differences between them. There were significant differences in the protective effects of various Shenmai injections on doxorubicin and oxidative stress-induced cardiomyocyte injury. Moreover, the protective effects of Shenmai injection with Zhejiang *O. japonicas* as the raw material are significantly better than those of Shenmai injections with Sichuan *O. japonicas*, consistent with our previous research results.

## Data Availability

Our experimental data have been presented through the figures and Supplementary Materials. These data support this study.
