# Supplementary material for: Comparison of Protective Effects of Shenmai Injections Produced by Medicinal Materials from Different Origins on Cardiomyocytes
Source: Evid Based Complement Alternat Med. 2022 Mar 18;2022:7205476. doi: 10.1155/2022/7205476 (PMC8956391; doi:10.1155/2022/7205476)
Supplement: Supplementary Materials — The data of one-way ANOVA can be found in the supplementary files. [file 7205476.f1.docx]

One-Way ANOVA of five Shenmai injections by Microsoft Excel.

**Figure 1b**

| Anova: Single Factor | |  |  |  |  |  |
| --- | --- | --- | --- | --- | --- | --- |
| SUMMARY |  |  |  |  |  |  |
| Groups | Count | Sum | Average | Variance |  |  |
| SM1 | 3 | 347.3617 | 115.7872 | 22.98574 |  |  |
| SM2 | 3 | 419.0388 | 139.6796 | 223.5308 |  |  |
| SM3 | 3 | 377.7195 | 125.9065 | 50.73 |  |  |
| SM4 | 3 | 350.3797 | 116.7932 | 39.48069 |  |  |
| SM5 | 3 | 390.8549 | 130.285 | 171.3241 |  |  |
| ANOVA |  |  |  |  |  |  |
| Source of Variation | SS | df | MS | F | P-value | F crit |
| Between Groups | 1182.26 | 4 | 295.5651 | 2.908811 | 0.077821 | 3.47805 |
| Within Groups | 1016.103 | 10 | 101.6103 |  |  |  |
| Total | 2198.363 | 14 |  |  |  |  |

**Figure 1c**

| Anova: Single Factor | |  |  |  |  |  |
| --- | --- | --- | --- | --- | --- | --- |
| SUMMARY | |  |  |  |  |  |
| Groups | Count | Sum | Average | Variance |  |  |
| SM1 | 3 | 424.2362 | 141.4121 | 352.9244 |  |  |
| SM2 | 3 | 466.4969 | 155.499 | 468.9821 |  |  |
| SM3 | 3 | 379.4297 | 126.4766 | 8.036653 |  |  |
| SM4 | 3 | 349.0041 | 116.3347 | 4.483398 |  |  |
| SM5 | 3 | 350.9165 | 116.9722 | 6.826743 |  |  |
| ANOVA |  |  |  |  |  |  |
| Source of Variation | SS | df | MS | F | P-value | F crit |
| Between Groups | 3421.047 | 4 | 855.2616 | 5.083259 | 0.016946 | 3.47805 |
| Within Groups | 1682.506 | 10 | 168.2506 |  |  |  |
|  |  |  |  |  |  |  |
| Total | 5103.553 | 14 |  |  |  |  |

**Figure 1d**

| Anova: Single Factor | |  |  |  |  |  |
| --- | --- | --- | --- | --- | --- | --- |
| SUMMARY | |  |  |  |  |  |
| Groups | Count | Sum | Average | Variance |  |  |
| SM1 | 3 | 360.8665 | 120.2888 | 52.33573 |  |  |
| SM2 | 3 | 415.8685 | 138.6228 | 61.2988 |  |  |
| SM3 | 3 | 403.8099 | 134.6033 | 59.74112 |  |  |
| SM4 | 3 | 386.5082 | 128.8361 | 64.13954 |  |  |
| SM5 | 3 | 378.9353 | 126.3118 | 161.6116 |  |  |
| ANOVA |  |  |  |  |  |  |
| Source of Variation | SS | df | MS | F | P-value | F crit |
| Between Groups | 613.3517 | 4 | 153.3379 | 1.920918 | 0.183461 | 3.47805 |
| Within Groups | 798.2535 | 10 | 79.82535 |  |  |  |
| Total | 1411.605 | 14 |  |  |  |  |

**Figure 1f**

| Anova: Single Factor |  |  |  |  |  |  |
| --- | --- | --- | --- | --- | --- | --- |
| SUMMARY |  |  |  |  |  |  |
| Groups | Count | Sum | Average | Variance |  |  |
| SM1 | 3 | 247.638 | 82.546 | 31.3857 |  |  |
| SM2 | 3 | 180.962 | 60.32067 | 169.6301 |  |  |
| SM3 | 3 | 222.28 | 74.09333 | 50.72706 |  |  |
| SM4 | 3 | 249.621 | 83.207 | 18.82136 |  |  |
| SM5 | 3 | 209.146 | 69.71533 | 88.3171 |  |  |
| ANOVA |  |  |  |  |  |  |
| Source of Variation | SS | df | MS | F | P-value | F crit |
| Between Groups | 1089.874 | 4 | 272.4686 | 3.796082 | 0.039627 | 3.47805 |
| Within Groups | 717.7626 | 10 | 71.77626 |  |  |  |
| Total | 1807.637 | 14 |  |  |  |  |

**Figure 3a**

| Anova: Single Factor | |  |  |  |  |  |
| --- | --- | --- | --- | --- | --- | --- |
| SUMMARY | |  |  |  |  |  |
| Groups | Count | Sum | Average | Variance |  |  |
| SM1 | 6 | 298.9104 | 49.8184 | 15.39408 |  |  |
| SM2 | 6 | 293.0993 | 48.84988 | 13.05484 |  |  |
| SM3 | 6 | 292.3729 | 48.72881 | 12.70307 |  |  |
| SM4 | 6 | 293.4625 | 48.91041 | 26.69888 |  |  |
| SM5 | 6 | 320.7022 | 53.45036 | 18.2961 |  |  |
| ANOVA |  |  |  |  |  |  |
| Source of Variation | SS | df | MS | F | P-value | F crit |
| Between Groups | 96.31288 | 4 | 24.07822 | 1.397508 | 0.263595 | 2.75871 |
| Within Groups | 430.7348 | 25 | 17.22939 |  |  |  |
| Total | 527.0477 | 29 |  |  |  |  |

**Figure 3c**

| Anova: Single Factor | |  |  |  |  |  |
| --- | --- | --- | --- | --- | --- | --- |
| SUMMARY | |  |  |  |  |  |
| Groups | Count | Sum | Average | Variance |  |  |
| SM1 | 6 | 1510.023 | 251.6705 | 129.2813 |  |  |
| SM2 | 6 | 1518.855 | 253.1425 | 196.7429 |  |  |
| SM3 | 6 | 1522.758 | 253.793 | 151.8765 |  |  |
| SM4 | 6 | 1509.885 | 251.6476 | 281.117 |  |  |
| SM5 | 6 | 1437.746 | 239.6243 | 135.9889 |  |  |
| ANOVA |  |  |  |  |  |  |
| Source of Variation | SS | df | MS | F | P-value | F crit |
| Between Groups | 824.5121 | 4 | 206.128 | 1.151545 | 0.355649 | 2.75871 |
| Within Groups | 4475.033 | 25 | 179.0013 |  |  |  |
| Total | 5299.545 | 29 |  |  |  |  |

**Figure 4b**

| Anova: Single Factor | |  |  |  |  |  |
| --- | --- | --- | --- | --- | --- | --- |
| SUMMARY | |  |  |  |  |  |
| Groups | Count | Sum | Average | Variance |  |  |
| SM1 | 3 | 125.06 | 41.68667 | 11.38923 |  |  |
| SM2 | 3 | 123.8 | 41.26667 | 11.44663 |  |  |
| SM3 | 3 | 129.72 | 43.24 | 5.9632 |  |  |
| SM4 | 3 | 134.94 | 44.98 | 1.5028 |  |  |
| SM5 | 3 | 109.11 | 36.37 | 11.9401 |  |  |
| ANOVA |  |  |  |  |  |  |
| Source of Variation | SS | df | MS | F | P-value | F crit |
| Between Groups | 124.6314 | 4 | 31.15786 | 3.688022 | 0.042837 | 3.47805 |
| Within Groups | 84.48393 | 10 | 8.448393 |  |  |  |
| Total | 209.1154 | 14 |  |  |  |  |

**Figure 5a**

| Anova: Single Factor | |  |  |  |  |  |
| --- | --- | --- | --- | --- | --- | --- |
| SUMMARY | |  |  |  |  |  |
| Groups | Count | Sum | Average | Variance |  |  |
| SM1 | 3 | 225.563 | 75.18768 | 17.49483 |  |  |
| SM2 | 3 | 306.3867 | 102.1289 | 21.64847 |  |  |
| SM3 | 3 | 232.7628 | 77.58761 | 5.466019 |  |  |
| SM4 | 3 | 240.1949 | 80.06497 | 29.79339 |  |  |
| SM5 | 3 | 312.6575 | 104.2192 | 15.98461 |  |  |
| ANOVA |  |  |  |  |  |  |
| Source of Variation | SS | df | MS | F | P-value | F crit |
| Between Groups | 2394.28 | 4 | 598.5701 | 33.1114 | 9.63E-06 | 3.47805 |
| Within Groups | 180.7746 | 10 | 18.07746 |  |  |  |
|  |  |  |  |  |  |  |
| Total | 2575.055 | 14 |  |  |  |  |

| Anova: Single Factor | |  |  |  |  |  |
| --- | --- | --- | --- | --- | --- | --- |
| SUMMARY | |  |  |  |  |  |
| Groups | Count | Sum | Average | Variance |  |  |
| SM1 | 3 | 174.6209 | 58.20695 | 0.125863 |  |  |
| SM2 | 3 | 190.9575 | 63.6525 | 28.6497 |  |  |
| SM3 | 3 | 156.9697 | 52.32324 | 33.06576 |  |  |
| SM4 | 3 | 186.0012 | 62.0004 | 130.0156 |  |  |
| SM5 | 3 | 219.6777 | 73.22591 | 24.65102 |  |  |
| ANOVA |  |  |  |  |  |  |
| Source of Variation | SS | df | MS | F | P-value | F crit |
| Between Groups | 710.1263 | 4 | 177.5316 | 4.099885 | 0.032021 | 3.47805 |
| Within Groups | 433.016 | 10 | 43.3016 |  |  |  |
| Total | 1143.142 | 14 |  |  |  |  |

**Figure 5b**

| Anova: Single Factor | |  |  |  |  |  |
| --- | --- | --- | --- | --- | --- | --- |
| SUMMARY | |  |  |  |  |  |
| Groups | Count | Sum | Average | Variance |  |  |
| SM1 | 3 | 588.7478 | 196.2493 | 196.9452 |  |  |
| SM2 | 3 | 304.4532 | 101.4844 | 319.1835 |  |  |
| SM3 | 3 | 558.9487 | 186.3162 | 240.9547 |  |  |
| SM4 | 3 | 549.2204 | 183.0735 | 53.52904 |  |  |
| SM5 | 3 | 268.37 | 89.45667 | 193.8574 |  |  |
| ANOVA |  |  |  |  |  |  |
| Source of Variation | SS | df | MS | F | P-value | F crit |
| Between Groups | 31686.95 | 4 | 7921.738 | 39.43243 | 4.3E-06 | 3.47805 |
| Within Groups | 2008.94 | 10 | 200.894 |  |  |  |
| Total | 33695.89 | 14 |  |  |  |  |

| Anova: Single Factor | |  |  |  |  |  |
| --- | --- | --- | --- | --- | --- | --- |
| SUMMARY | |  |  |  |  |  |
| Groups | Count | Sum | Average | Variance |  |  |
| SM1 | 3 | 801.5165 | 267.1722 | 83.43389 |  |  |
| SM2 | 3 | 736.17 | 245.39 | 139.8155 |  |  |
| SM3 | 3 | 848.1211 | 282.707 | 154.2519 |  |  |
| SM4 | 3 | 742.9952 | 247.6651 | 371.2564 |  |  |
| SM5 | 3 | 610.2891 | 203.4297 | 189.4928 |  |  |
| ANOVA |  |  |  |  |  |  |
| Source of Variation | SS | df | MS | F | P-value | F crit |
| Between Groups | 10672.46 | 4 | 2668.115 | 14.21856 | 0.000393 | 3.47805 |
| Within Groups | 1876.501 | 10 | 187.6501 |  |  |  |
| Total | 12548.96 | 14 |  |  |  |  |

**Figure 5d**

| Anova: Single Factor | |  |  |  |  |  |
| --- | --- | --- | --- | --- | --- | --- |
| SUMMARY | |  |  |  |  |  |
| Groups | Count | Sum | Average | Variance |  |  |
| SM1 | 3 | 79.52 | 26.50667 | 0.460433 |  |  |
| SM2 | 3 | 51 | 17 | 8.6401 |  |  |
| SM3 | 3 | 75.91 | 25.30333 | 0.382033 |  |  |
| SM4 | 3 | 75.05 | 25.01667 | 2.661033 |  |  |
| SM5 | 3 | 45.17 | 15.05667 | 6.505833 |  |  |
| ANOVA |  |  |  |  |  |  |
| Source of Variation | SS | df | MS | F | P-value | F crit |
| Between Groups | 339.8485 | 4 | 84.96212 | 22.77874 | 5.21E-05 | 3.47805 |
| Within Groups | 37.29887 | 10 | 3.729887 |  |  |  |
| Total | 377.1473 | 14 |  |  |  |  |

| Anova: Single Factor | |  |  |  |  |  |
| --- | --- | --- | --- | --- | --- | --- |
| SUMMARY | |  |  |  |  |  |
| Groups | Count | Sum | Average | Variance |  |  |
| SM1 | 3 | 141.36 | 47.12 | 2.6053 |  |  |
| SM2 | 3 | 128.07 | 42.69 | 4.0603 |  |  |
| SM3 | 3 | 145.45 | 48.48333 | 2.573033 |  |  |
| SM4 | 3 | 142.77 | 47.59 | 4.4401 |  |  |
| SM5 | 3 | 110.25 | 36.75 | 6.8047 |  |  |
| ANOVA |  |  |  |  |  |  |
| Source of Variation | SS | df | MS | F | P-value | F crit |
| Between Groups | 286.8435 | 4 | 71.71087 | 17.5046 | 0.000164 | 3.47805 |
| Within Groups | 40.96687 | 10 | 4.096687 |  |  |  |
|  |  |  |  |  |  |  |
| Total | 327.8103 | 14 |  |  |  |  |
